# Haplotype dependent association of rs7927894 (11q13.5) with atopic dermatitis and chronic allergic rhinitis: A study in ECAP cohort

**DOI:** 10.1371/journal.pone.0183922

**Published:** 2017-09-08

**Authors:** Joanna Kinga Ponińska, Bolesław Samoliński, Aneta Tomaszewska, Filip Raciborski, Piotr Samel-Kowalik, Artur Walkiewicz, Agnieszka Lipiec, Barbara Piekarska, Edyta Krzych-Fałta, Andrzej Namysłowski, Grażyna Kostrzewa, Andrzej Pawlik, Monika Jasek, Andrzej Wiśniewski, Piotr Kuśnierczyk, Sławomir Majewski, Rafał Płoski

**Affiliations:** 1 Department of Medical Biology, Institute of Cardiology, Warsaw, Poland; 2 Department of Prevention of Environmental Hazards and Allergology, Medical University of Warsaw, Warsaw, Poland; 3 Department of Medical Genetics, Medical University of Warsaw, Warsaw, Poland; 4 Department of Physiology, Pomeranian Medical University, Szczecin, Poland; 5 Laboratory of Immunogenetics, Ludwik Hirszfeld Institute of Immunology and Experimental Therapy, Polish Academy of Sciences, Wroclaw, Poland; 6 Department of Dermatology and Venereology, Medical University of Warsaw, Warsaw, Poland; Hospital Israelita Albert Einstein, BRAZIL

## Abstract

The T allele of rs7927894 (at 11q13.5) was associated with atopic dermatitis and other allergic diseases. Our purpose was to replicate the association with allergic phenotypes and explore the role of rs7927894 in predisposing to persistent allergic rhinitis and atopic asthma. We also wanted to explore if other SNPs at 11q13.5 contributed to effect of rs7927894. We studied patients with atopic dermatitis (N = 270), atopic asthma (N = 486), persistent allergic rhinitis (N = 589) and controls matched for age, sex and region (N = 540, N = 372 and N = 1178, respectively). We found that rs7927894 T was associated with atopic dermatitis (OR = 1.39, CI: 1.12–1.73, P = 0.003) and independently with persistent allergic rhinitis (OR = 1.24, CI:1.07–1.43, P = 0.0043, P_corrected_ = 0.013) but not atopic asthma. Analysis of additional tagging SNPs (rs7930763, rs2513517, rs7125552) showed that effect of rs7927894 T was limited to haplotypes encoding G at rs7125552. In conclusion, rs7927894 T is associated not only with atopic dermatitis but also persistent allergic rhinitis. Since these effects are haplotype dependent rs7927894 alone does not account for the association between 11q13.5 and atopic dermatitis/persistent allergic rhinitis.

## Introduction

Allergy is the most common disease in developmental age. At present, the prevalence rate is almost 40% of the population in developed countries with trends for increase [1]. The strongest hypothesis explaining this high prevalence is related to the hygienic character style of life in neonatal age [2].

The phenotypes of allergic diseases are different according to age. During the first 3 years of life, atopic dermatitis (AD) is the most common but its incidence decreases tenfold in the following years [3]. Conversely, allergic rhinitis (AR), in particular the persistent form (pAR, see Materials and Methods for definition) remains widespread and will be common not only in childhood, but in the following decades. Allergic asthma (AA) is usually a natural consequence of AR in more than 30% of AR patients [4]. There is a strong connection between the upper and lower respiratory tract and AR and AA are commonly present in the same patients [5]. The reason for this is still being discussed but shared genetic factors are a possible cause [6].

Since the identification of loss-of-function variants at the filaggrin (*FLG*) locus as strong and widely replicated risk factors for AD [7] there is increasing evidence for the central role of epidermal barrier defects in predisposing to AD [8–10]. Interestingly, the *FLG* defects have also been associated with an increased risk for development of other allergic diseases such as allergic rhinitis (AR) and atopic asthma (AA) also in the absence of AD [10]. In particular, in a recent study in a Polish population we have found that two most frequent *FLG* null variants 2282del4 and R501X were associated not only with AD but also with AA and persistent allergic rhinitis (pAR) [11]. The association to AA was not secondary to association with AD whereas for pAR the data were not conclusive [11]. These observations suggest that epidermal barrier defects are likely to play a role in pathogenesis of not only AD but other allergies, in particular AA and pAR.

Recently, a large genome-wide association study showed that genetic variants at chromosome 11q13.5, in particular the minor allele of rs7927894 (i.e. T or A depending on a DNA molecule strand), were also associated with AD [12]. Whereas the precise identity of gene(s) responsible for this association is not known it was noted that the same genetic variant had also been associated with Crohn disease [13]. Since Crohn disease is considered to be caused by defect(s) in epithelial barrier [14] it has been suggested that the rs7927894 may exert its effect through impairment of epithelial function [12].

The association between AD and rs7927894 was confirmed by multiple studies in populations of Caucasian ancestry [15–18]. There are also several reports about the role of 11q13.5 variations in predisposing to other allergic phenotypes like allergic rhinitis [19], asthma [20], serum IgE levels in asthma [21], grass sensitization [19], atopic march [22], and eosinophilic esophagitis [23]. Interestingly, no association was found between variants in 11q13.5 and allergic diseases in Asian patients [24–26].

Our purpose was to replicate in a Polish population the association of the T allele of rs7927894 with AD and explore the role of this genetic variant in predisposing to other diseases, in particular pAR and AA which in our recent study were linked to epidermal barrier defects similar as AD [11]. We were also interested whether analysis of other SNPs in 11q13.5 region could reveal stronger association(s), including haplotype specific effects, than the one found with rs7927894.

## Materials and methods

### Ethics statement

The study was approved by Ethical Committee of Medical University of Warsaw. All ECAP subjects gave an informed written consent including specific consent to genetic testing.

### Subjects

The present study included a subset of participants of Epidemiology of Allergic Diseases in Poland (ECAP, http://www.ECAP.pl) study. ECAP is a population based study which included randomly selected subjects aged 20–44 y.o. as well as 6–7 y.o. and 13–14 y.o. as described previously [11].

As described previously [11] all subjects underwent examination including medical history, physical examination, spirometry, PNIF (Peak Nasal Inspiratory Flow) and skin prick tests with 15 allergens (Allergopharma): hazel, alder, birch, grasses/grain, rye, Artemisia, plantain, Alternaria, Cladosporium, molds I (Alternaria tenuis, Botrytis cinerea, Cladosporium herbarum, Culvularia lunata, Fusarium moniliforme, Helminthosporium), molds II (Aspergillus fumigatus, Mucor mucedo, Penicillium notatum, Rhizopus nigricans, Serpula lacrymans, Pullularia pullulans), Dermatophagoides pteronyssinus, Dermatophagoides farinae, dog, cat, negative control, histamine.

The clinical diagnoses of atopic asthma, persistent allergic rhinitis, (i.e. with symptoms present > 4 days a week and for > 4 consecutive weeks) and atopic dermatitis were based on the International Global Initiative for Asthma (GINA) guidelines [27], ARIA criteria [28, 29], and criteria of Hanifin and Rajka [30], for asthma, allergic rhinitis and atopic dermatitis, respectively.

We studied subjects with AD (N = 270), AA (N = 186) and pAR (N = 589). For each of these groups a specific control group consisting of healthy subjects (i.e. subjects without symptoms or history of allergic/non-allergic diseases) was selected to be matched with the patients regarding age category, sex and clinical center where the patient was examined (corresponding to region of inhabitance). For each group twice as many controls as affected subjects were selected (i.e. 540, 372 and 1 178 controls for AD, AA and pAR, respectively). These three groups of controls are referred to as “the matched controls”. In total, these controls included 1 258 distinct subjects and were also used after pooling (“the pooled ECAP controls”). Detailed demographic data of studied ECAP subjects is provided in S1 Table.

In order to increase power to detect association with AA we also studied two independently recruited groups of adult patients and geographically matched healthy non-atopic controls from Szczecin (Pomeranian Medical University, N_pat._ = 208, N_contr._ = 200) and Wroclaw, (Department of Internal Medicine and Allergology of Wroclaw Medical University, N_pat._ = 92, N_contr._ = 87). Mean age (SD) of patients was 45 (17.9) and 40 (16) for Szczecin and Wroclaw, respectively whereas percentage of females was 57% and 63%, respectively. Both cohorts were described previously [31].

### Genetic analysis

Genomic DNA was extracted from whole blood. Genotyping was performed by TaqMan allelic discrimination assays (Assay on demand, cat. no. 4332072 *Life Technolgies*). PCR was performed using qPCR MasterMix Plus (cat. no. RT-QP2X-03+WOULR *Life Technolgies*) on ABI PRISM 7500 (*Applied Biosystems*) in a volume of 10 μl using default thermal profile. The genotype distribution did not deviate from Hardy-Weinberg equilibrium (0.15<P<0.97 for all studied groups).

To select additional SNPs we queried Ensembl database (http://www.ensembl.org) and retrieved all SNPs showing in Caucasians LD with rs7927894 of r^2^>0.5 using both HapMap [32] and 1000 Genomes [33] project data. From those SNPs we selected a set of tags. The characteristics of initially selected SNPs and the tagging SNPs which were finally analyzed is shown in S2 Table (all SNPs were tagged with r^2^>0.8 apart from rs4494327 which was tagged at r^2^ = 0.71). Pairwise linkage disequilibria among tested SNPs as found in our population are shown in S3 Table.

### Statistical analysis

Statistical significance of differences in allele frequency was assessed with chi square test whereas differences in distribution of genotypes were analyzed with logistic regression assuming additive model. The strength of association was estimated by calculating Odds Ratio (OR) with 95% confidence interval (CI). Stratified analysis of three AA cohorts was done with Mantel-Haenszel test. These calculation were performed using *SPSS* package.

A correction for number of comparisons was done by Bonferroni method. Whenever this correction was applied the P values are shown as ‘P_corrected_’. The correction factor was 11 and was determined by the following considerations. In the first part of the study we tested for allelic association between rs7927894 and 3 phenotypes (AD, pAR, AA). Next, in AD cohort we analyzed 3 additional SNPs which together with rs7927894 allowed to define 8 haplotypes whose distribution was compared between the affected and control group. Thus, in total 8+3 = 11 comparisons were made. Testing for rs7927894 effect in pAR with and without AD was not Bonferroni corrected since it was an exploratory analysis. Similarly, testing for rs7927894-rs7125552 haplotypes in pAR was not Bonferroni corrected as the analysis was driven by specific hypothesis. Hardy-Weinberg equilibrium and haplotype analyses were performed by PLINK [34].

Our study had the power of 0.8 to detect the association (at alpha 0.05) between AD and the T allele of rs7927894 of the magnitude originally reported [12]. The power to detect association with pAR and AA in ECAP cohort was, respectively, 0.9 and 0.6 whereas power to detect association with AA in three cohorts was 0.86. Power calculations were performed by *Statistica* package and PS program (for Mantel-Haenszel analysis) [35] http://biostat.mc.vanderbilt.edu/twiki/bin/view/Main/PowerSampleSize.

## Results

### The T allele of rs7927894 is associated with atopic dermatitis (AD) and persistent allergic rhinitis (pAR)

We found that the T allele of rs7927894 was overrepresented among AD and pAR patients compared to the matched controls (Table 1). Analysis of genotype distribution under an additive model also showed associations: OR = 1.39 (CI:1.12–1.73), P = 0.003, P_corrected_ = 0.009 and OR = 1.24 (1.07–1.43), P = 0.0043, P_corrected_ = 0.013, for AD and pAR, respectively).

**Table 1:** Distribution of alleles and genotypes of rs7927894 among patients and controls.

Disease	Genotypes								Allele freq.					
	Affected				Controls				Affected		Controls		OR _allelic_ (CI)	P (P_corr._)
	N	CC	CT	TT	N	CC	CT	TT	N	‘T’	N	‘T’		
		n (%)	n (%)	n (%)		n (%)	n (%)	n (%)		n (%)		n (%)		
AD	270	101 (37)	128 (47)	41 (15)	540	254 (47)	233 (43)	53 (10)	540	210 (39)	1080	339 (31)	1.39 (1.12–1.73)	0.003 (0.033)
pAR	589	236 (40)	265 (45)	88 (15)	1178	538 (46)	512 (44)	128 (11)	1178	441 (37)	2356	768 (33)	1.24 (1.07–1.43)	0.0043 (0.047)
pAR—AD	521	211 (41)	237 (46)	73 (14)	See above	See above	See above	See above	1042	383 (37)	See above	See above	1.20 (1.03–1.40)	0.018
pAR + AD	68	25 (37)	28 (41)	15 (22)	See bove	See above	See above	See above	136	58 (43)	See above	See above	1.54 (1.08–2.18)	0.015
AA ECAP	186	80 (43)	80 (43)	26 (14)	372	170 (46)	155 (42)	47 (13)	372	132 (35)	744	249 (34)	1.09 (0.84–1.42)	0.50
AA Szcz.	208	78 (38)	94 (45)	36 (17)	200	82 (41)	98 (49)	20 (10)	416	166 (40)	400	138 (35)	1.26 (0.95–1.68)	0.11
AA Wroc.	92	33 (36)	40 (43)	19 (21)	87	25 (29)	49 (56)	13 (15)	184	78 (42)	174	75 (43)	0.97 (0.64–1.48)	0.89
Combined	486	191 (40)	214 (44)	81 (17)	659	277 (42)	302 (46)	80 (12)	972	376 (39)	1318	462 (35)	1.17 (0.98–1.39)	0.075

N–total number of allele/subjects in a group; n–number of particular alleles/genotypes; P_corr._—P values corrected for the number of comparisons (N = 11) by Bonferroni method

Exploratory analysis of distribution of alleles and genotypes of rs7927894 among pAR patients stratified by presence of AD showed that the association with pAR most likely was not secondary to the association with AD. As can be seen from Table 1 the increase in prevalence of the allele in comparison to healthy controls was comparable among those with pAR without and with AD (OR = 1.20; P = 0.018 and OR = 1.54; P = 0.015, respectively). Similar results were obtained when genotype distribution was analyzed: OR = 1.20 (CI: 1.03–1.39), P = 0.02 and OR = 1.52 (CI: 1.08–2.16), P = 0.019, respectively for those without and with AD.

We did not observe an association with AA in any of the analyzed groups or in the combined analysis (Table 1).

### Analysis of other SNPs in the 11q13.5 region shows that the effect rs7927894 T is haplotype dependent

Analysis of additionally typed SNPs (Table 2) revealed a single trend for association with AD (rs2513517, P = 0.039) which by conditional analysis was shown to be secondary to effect of rs7927894 (P value for independent effect of rs2513517 = 0.98).

**Table 2:** Distribution of genotypes of 11q13.5 SNPs among subjects with AD and controls.

	N	Minor allele	Affected	Control	OR	CI		P
rs7927894	810	T	41/128/101	53/233/254	1.39	1.12	1.73	0.0028*
rs7930763	799	A	75/126/66	118/264/150	1.20	0.98	1.48	0.08
rs2513517	806	T	72/136/60	120/263/155	1.24	1.01	1.53	0.039
rs7125552	794	A	28/113/122	47/229/255	1.08	0.86	1.36	0.49

*P_corrected_ = 0.031

Further analysis showed that in the studied groups there were 8 distinct haplotypes with frequencies higher or equal to 0.01 two of which encoded allele T at rs7927894 (the TATA and TATG, haplotypes Table 3). Interestingly, whereas one of these haplotypes (TATG) showed association with AD (OR = 1.92, P = 0.00021, P_corrected_ = 0.002) the other (TATA) did not (OR = 1.12, P = 0.36, Table 3). The lack of effect of the TATA haplotype was not due to type II error possibly caused by its low frequency as this haplotype was approximately two times more prevalent than TATG (Table 3).

**Table 3:** Distribution of 4 locus (rs7927894, rs7930763, rs2513517, rs7125552) or 2 locus (rs7927894, rs7125552) haplotypes among subjects with AD and controls.

Disease	Haplotype	Affected	Control	OR	P value
AD	**TATA**	0.26	0.24	1.12	0.36
	CGTA	0.04	0.04	0.97	0.9
	CGCA	0.01	0.01	0.90	0.83
	**TATG**	0.12	0.07	1.92	0.00021*
	CATG	0.02	0.02	0.83	0.62
	CGTG	0.08	0.09	0.89	0.56
	CACG	0.10	0.13	0.76	0.01
	CGCG	0.36	0.39	0.87	0.2
AD	**TA**	0.26	0.24	1.12	0.37
	CA	0.06	0.06	0.94	0.78
	**TG**	0.13	0.07	1.98	0.000095#
	CG	0.55	0.63	0.73	0.0034
pAR	**TA**	0.27	0.24	1.13	0.15
	CA	0.05	0.06	0.92	0.593
	**TG**	0.10	0.08	1.32	0.023
	CG	0.58	0.62	0.84	0.019

*P_corrected_ = 0.002

#P_corrected_ = 0.001

We also found that the TATG haplotype fully explained the association with AD observed in our study (after controlling for effect of this haplotype the omnibus test of association at 11q13.5 was not significant (P = 0.27)). In contrast, controlling for the effect of the TATA haplotype did not affect the overall association (P = 0.00016)

Next we studied whether the haplotype specific effect was also present in pAR. Since in addition to rs7927894 the haplotypes TATA and TATG differ only in rs7125552 we limited additional typing to this SNP. Analysis of rs7927894-rs7125552 haplotype distribution showed that the TG (OR = 1.32, P = 0.023) but not the TA haplotype (OR = 1.13, P = 0.15, Table 3) was associated. In further similarity to AD we found that the TG but not TA haplotype explained association at 11q13.5 (respective P values: 0.20 and, 0.038).

## Discussion

In the present study we replicated the association between T allele of rs7927894 and AD in a Polish Caucasian population. We also showed for the first time that this genetic variant is independently associated with pAR. The independent association between self-reported AR and another SNP in this locus (rs2155219) in moderate linkage disequilibrium with rs7927894 has been reported previously[19]. We also provide evidence that both in AD and pAR rs7927894 T alone does not explain the association at 11q13.5 since its effect depends on genotype of rs7125552.

Our study is the subsequent report in Caucasians in which the association between T allele of rs7927894 and AD was observed [12, 15, 16]. The consistency of findings in Caucasians contrasts with Asian reports in a Japanese, Chinese, and Korean populations where no association between AD and rs7927894 or other variants in locus 11q13.5 was observed [24–26]. Our data indicating that effect of rs7927894 is haplotype dependent is consistent with findings in Asians as it is likely that patterns of linkage disequilibrium at 11q13.5 are substantially different between Caucasians and Asians.

The functionally relevant variant responsible for association between rs7927894 and AD has not been unequivocally identified. rs7927894 is located in an intergenic region between C11orf30/EMSY (chromosome 11 open reading frame 30 encoding EMSY protein) and LRRC32/GARP (leucine rich repeat containing 32/glycoprotein A repetitions predominant) genes. The EMSY protein interacts with BRCA2 and thus may have a role in epithelial differentiation. Involvement of rs7927894 in epithelial differentiation would be consistent with association of the same SNP also with Crohn disease which is regarded as an archetypal inflammatory barrier disease [13, 14]. On the other hand, *LRRC32* may be a better candidate as a susceptibility locus since GARP functions as a receptor specific to T(reg) that binds latent TGF-beta controlling expression of FOXP3 and the regulatory phenotype [36]. This broad function in immune system is also consistent with rs7927894 association with Crohn disease as well as our findings showing that susceptibility conferred by this variant extends to pAR.

The fact that effect of rs7927894 is haplotype dependent suggests that this SNP may not be primarily involved in disease pathogenesis but is a marker of another functionally important variant. This conclusion is consistent with recent findings of Manz et al. who proposed that rare variants in GARP are responsible for the association between AD and 11q13.5 [37].

We did not find association between rs7927894 and AA which contrasts with association between AA and *FLG* null variants observed in the same cohort [11]. This may further suggest that rs7927894 may be associated with disease in a mechanism different from *FLG* variants, in particular it may be in LD with some rare coding variant(s) in GARP, as proposed by Manz et al [37]. Alternatively, as the two SNPs are ~90 kb apart it is possible that they both influence chromatin structure affecting translation. Functional studies as well as sequencing of coding regions of GARP on rs7927894 T-rs7125552 G haplotypes should be performed to determine the mechanism of association observed by us.

In conclusion, we replicated the association between the T allele of rs7927894 and AD in a Polish Caucasian population and showed that this genetic variant is also associated with pAR. In both AD and pAR the effect of rs7927894 T is haplotype dependent and occurs only if G is present at rs7125552 suggesting that rs7927894 alone does not account for the association between 11q25 and AD/pAR.

## Supporting information

S1 Table Demographic data for subjects from ECAP cohort. y.o.–years old.
(DOCX)

S2 Table Characteristics of SNPs analyzed in the study. (DOCX)

S3 Table Pairwise linkage disequlibria (r^2^) among tested SNPs in affected subjects and controls. (DOCX)
