# Supplementary material for: Haplotype dependent association of rs7927894 (11q13.5) with atopic dermatitis and chronic allergic rhinitis: A study in ECAP cohort
Source: PLoS One. 2017 Sep 8;12(9):e0183922. doi: 10.1371/journal.pone.0183922 (PMC5590850; doi:10.1371/journal.pone.0183922)
Supplement: S1 Table — y.o.–years old. (DOCX) [file pone.0183922.s001.docx]

**S1 Table.** **Demographic data for subjects from ECAP cohort**

| Group | Females | Children 6-7 y.o. | Children 6-7 y.o. | Adults | | Self-declared history of allergy in the family* | Other comordibitis |
| --- | --- | --- | --- | --- | --- | --- | --- |
|  | n (%) | n (%) | n (%) | n (%) | mean age in years (SD) | n (%) | n (%) |
| AD | 154 (57) | 194 (36) | 195 (36) | 151 (28) | 30.9 (7.4) | 146 (54)^1)^ | 36 (13)^4)^ |
| AD control | 308 (57) | 97 (36) | 104 (38) | 69 (26) | 31.0 (7.3) | 175 (32) | 0 (0) |
| AA | 78 (42) | 41 (22) | 56 (30) | 89 (48) | 30.0 (7.1) | 83 (46)^2)^ | 26 (14)^5)^ |
| AA control | 156 (42) | 84 (23) | 114 (31) | 174 (47) | 31.4 (7.3) | 99 (27) | 0 (0) |
| pAR | 277 (47) | 133 (23) | 179 (30) | 277 (47) | 29.8 (7.2) | 256 (44)^3)^ | 71 (12)^6)^ |
| pAR control | 554 (47) | 280 (24) | 348 (30) | 550 (47) | 31 (7.5) | 359 (31) | 0 (0) |

y.o. –years old;

* grandparents, parents, siblings

1) AD vs. AD control: OR=2.56 (95% CI: 1.82 – 3.31), p<0.0001

2) AA vs. AA control: OR=2.22 (95% CI: 1.54 – 3.22), p<0.0001

3) pAR vs. pAR control: OR=1.75 (95% CI: 1.43 – 2.15), p<0.0001

4) AD vs. AD control: p=0.0003

5) AA vs. AA control: p=0.0008

6) pAR vs. pAR control: p<0.0001
