# Supplementary material for: Haplotype dependent association of rs7927894 (11q13.5) with atopic dermatitis and chronic allergic rhinitis: A study in ECAP cohort
Source: PLoS One. 2017 Sep 8;12(9):e0183922. doi: 10.1371/journal.pone.0183922 (PMC5590850; doi:10.1371/journal.pone.0183922)
Supplement: S2 Table — (DOCX) [file pone.0183922.s002.docx]

**S2 Table.** **Characteristics of SNPs analyzed in the study**

| Tag SNP | SNP | Location | Distance from s7927894 (bp) | r^2^ vs. Tag SNP | r^2^ vs.rs7927894 | D' vs.rs7927894 | Located in gene(s) |
| --- | --- | --- | --- | --- | --- | --- | --- |
| rs7927894 | rs7130588 | 76 270 683 | 30633 | 0.96 | 0.96 | 1 | - |
|  | rs6592645 | 76 271 005 | 30311 | 0.90 | 0.90 | 1 | - |
|  | rs7926914 | 76 301 255 | 61 | 0.97 | 0.97 | 1 | - |
|  | rs7927997 | 76 301 375 | 59 | 0.96 | 0.96 | 1 | - |
| rs7125552 | rs7125552 | 76 211 094 | 90222 | tag | 0.50 | 0.78714 | C11orf30 |
|  | rs7115331 | 76 218 590 | 82726 | 0.88 | 0.62 | 0.95118 | C11orf30 |
| rs2513517 | rs2508746 | 76 270 950 | 30366 | 0.95 | 0.55 | 0.92296 | - |
|  | rs2513517 | 76 271 261 | 30055 | tag | 0.61 | 1 | - |
|  | rs2508747 | 76 271 689 | 29627 | 1.00 | 0.60 | 1 | - |
|  | rs6592652 | 76 285 579 | 15737 | 0.80 | 0.51 | 1 | - |
| rs7930763 | rs2212434 | 76 281 593 | 19723 | 0.90 | 0.72 | 0.95911 | *-* |
|  | rs61893460 | 76 291 154 | 10162 | 0.93 | 0.75 | 0.95994 | *-* |
|  | rs7126418 | 76 292 573 | 8743 | 0.90 | 0.71 | 0.92095 | *-* |
|  | rs7110818 | 76 292 575 | 8741 | 0.90 | 0.71 | 0.92095 | *-* |
|  | rs4494327 | 76 294 836 | 6480 | 0.71 | 0.65 | 0.88055 | - |
|  | rs11236791 | 76 295 598 | 5718 | 0.93 | 0.75 | 0.95994 | *-* |
|  | *rs10160518* | 76 296 671 | 4645 | 0.84 | 0.73 | 1 | *-* |
|  | *rs2155219* | 76 299 194 | 2122 | 0.84 | 0.73 | 1 | *-* |
|  | rs11236797 | 76 299 649 | 1667 | 0.93 | 0.75 | 0.95994 | *-* |
|  | rs7931483 | 76 302 067 | 751 | 0.83 | 0.59 | 0.8072 | RP11-672A2.7 processed pseudogene sim. to nucleolin |
|  | *rs7930763* | 76 302 073 | 757 | tag | 0.68 | 0.88358 | *RP11-672A2.7* |
