# Supplementary material for: Haplotype dependent association of rs7927894 (11q13.5) with atopic dermatitis and chronic allergic rhinitis: A study in ECAP cohort
Source: PLoS One. 2017 Sep 8;12(9):e0183922. doi: 10.1371/journal.pone.0183922 (PMC5590850; doi:10.1371/journal.pone.0183922)
Supplement: S3 Table — (DOCX) [file pone.0183922.s003.docx]

**S3 Table.** **Pairwise linkage disequlibria (r^2^) among tested SNPs in affected subjects and controls**

| Group |  | rs7927894 | rs7930763 | rs2513517 | rs7125552 |
| --- | --- | --- | --- | --- | --- |
| AD | rs7927894 | * | 0.62 | 0.51 | 0.35 |
|  | rs7930763 | 0.62 | * | 0.28 | 0.17 |
|  | rs2513517 | 0.51 | 0.28 | * | 0.33 |
|  | rs7125552 | 0.35 | 0.17 | 0.33 | * |
| Controls | rs7927894 | * | 0.52 | 0.53 | 0.49 |
|  | rs7930763 | 0.52 | * | 0.23 | 0.23 |
|  | rs2513517 | 0.53 | 0.23 | * | 0.44 |
|  | rs7125552 | 0.49 | 0.23 | 0.44 | * |
